# The Reply by Dr. Hume

**Published:** 1914-06-27

**Authors:** 


### The Reply by Dr. Hume.

Dr. Hume, in rising to reply to the debate, said that he was extremely gratified at the way in which his paper had been spoken of, and thought that there had been perhaps a little misconception as to the drift of the paper. Mr. Straker had criticised the paper on the ground that he (Dr. Hume) had not sufficiently emphasised those classes who should contribute to the hospitals. If they referred to his paper they would find that he said that in the first place one class alone could not, and ought not, to maintain an institution in which all had a vital concern. He had been rather interested to notice that one speaker after another speaker had risen and begun when they spoke to give a warm eulogium to the voluntary system, and then after that they proceeded to give it away. His friend, Sir Riley Lord, with whom it had been his delight and advantage to co-operate in hospital work for very many years, was an admirable example of the case in point. He claimed that Sir Riley was still in favour of the voluntary system, and that he was a wholehearted supporter of his paper. He had advocated State assistance, as there was such a need for increased hospital accommodation. It was his contention that if they began to claim State or municipal assistance for this or that, they were giving the whole case away.

Sir Henry Burdett: Municipal aid has been going on in this country for quite half a century, but we have not got yet a State hospital.

Dr. Hume: We have no municipal aid here.

Sir Henry Burdett: I am speaking of the whole country.

Dr. Hume said that all he could do was to point out that to depart further and further from the voluntary system and to accept State or municipal support brought with it inevitably State or municipal control. But the drift of his paper from beginning to end was contained in what he said at the beginning, that he intended to give a review of the position, and that he proposed to end with a forecast. He hoped that in the future they would have brought to them a great deal of increasing outside or State support, and that this would lead to what he hoped would be municipal control. As to Sir Henry Burdett's criticism, he had most cordially welcomed that and practically accepted it all, with the exception of one part. He knew perfectly well that after reading the paper he should come under the destructive criticism of Sir Henry Burdett, but he had learned a very great deal from him. He did not agree with him, however, when he said that the more scientific they became the more cruel they were apt to become, or that they were likely to become more indifferent to the feelings and interests of the patients. He knew that Sir Henry had had far more experience and acquaintance with hospitals in other countries than he (Dr. Hume) could ever hope to have. They approached the subject from two rather different standpoints. He had done work necessarily from the professional point of view, and his acquaintance with Continental hospitals began a very long time ago, when he spent a year in the German hospitals. But he had visited Continental hospitals since then. He visited only the other day a large provincial hospital in France, which was fully equipped from the scientific point of view, and was in conjunction with the university with regard to laboratories and such-like. Certainly, he could not gather there that the patients were worse off for science. Undoubtedly in many of the Continental hospitals the patients were in a poor position in comparison with ourselves in the voluntary hospitals. He did not think that there would be many to agree with the suggestion that the increase of science led to the neglect of the patients.

The proceedings of the Conference were then adjourned until the following day. Members of the house committee of Newcastle Infirmary entertained to luncheon those who were at the Conference, which was attended by the leading citizens, including the Lord Mayor and the Sheriff.

In the afternoon the Lord Mayor and the Lady Mayoress gave a garden-party in the Leazes Park, which adjoins the infirmary grounds, and there was a very large attendance, despite the fact that there had been a sharp thunderstorm and a copious fall of rain just before the time fixed for the reception. Visits were also made during the day to the wards and various departments of the Royal Victoria Infirmary.

